# Medical Extracts

**Published:** 1883-07

**Authors:** 


### Medical Extracts.

---

### The First Nephro-lithotomy. (?)

*The Way of Cutting for the Stone in the Kidneys, by* Mr. Charles Bernard. *Phil. Trans.*, No. 223, pp. 333.

*Mr. Hobson*, who was Consul for the *English* at *Venice*, having been long afflicted with the Stone in the Kidney, was at length attacked with a Fit of that Duration and Violence, that it reduced him almost to Despair; and finding no relief from any Means that had been used, and being under the greatest Extremity of Pain imaginable, he addressed himself to *Dominicus de Marchettis*, a famed and experienced Physician at *Padua*, begging of him that he would be pleased to cut the Stone out of his Kidney, being persuaded that there remained no other Method capable of relieving him; adding, that he was not insensible of the Danger, but that Death itself was infinitely more eligible than a Life in that Misery. *Marchetti* seemed very desirous to have declined it, representing not only the extreme Hazard, but, as he feared, the Impracticableness of the Operation; that it was what he had never attempted, and that, to proceed to it, was in effect to destroy him. But *Marchetti* was at length prevailed upon, by his Importunity, to undertake it; and having prepared him as he thought proper, he began with his Knife, cutting gradually upon the Region of the Kidney affected, till the Blood disturbed the Operation, so that he could not finish it at that Attempt; wherefore dressing up the Wound till the next Day, he then repeated and accomplished it, by cutting into the Body of the Kidney, and taking thence two or three small Stones, he dressed it up again; from this Instant he was freed from the Severity of the Pain, and in a reasonable Time he was able to walk about his Chamber, having been in no Danger, either from a Flux of Blood or Fever. *Marchetti* continued to dress the Wound for a considerable Time, but was not able to close it up, it soon becoming fistulous, from the continual Flux of Urine through the *Sinus*. But being in all other Respects restored to his former Health and Vigour, and the Matter discharged being little in Quantity, he took Leave of the Professor, and returned to *Venice* under the Care and Management of his Wife, who, one Morning as she was dressing the Sore, imagined she felt something hard and rugged as she wiped it; upon which, examining a little more carefully with her Bodkin, she found it to be a Stone of the Figure and Magnitude of a Date-stone; which being removed, he never after complained of the least Uneasiness in that Part. The Matter discharged was but little in Quantity, but always diluted with, and smelling strong of Urine. The Orifice would sometimes close for three or four Days together, and then the Matter would make its Way through the common Passages with the Urine, yet without any Difficulty or Pain. He applied nothing to the Orifice but a clean linnen Rag. He was able to perform all the Functions of Life, and to undergo any Fatigue, though upwards of 50 Years of Age.—*Mihles' Medical Essays.* 1745.

### Free Removal of Mammary Cancer.

Mr. Mitchell Banks (*Liverpool Medico-Chirurgical Journal*, January, 1883) strongly recommends in every case of Cancer of the Breast a removal not only of the whole breast with its superimposed skin, but also of all the axillary glands, even though, to the eye and touch, they may not seem to be affected. He says:—"Surgeons, as a rule, do not remove cancers of the breast. They persuade their patients that they do, and they almost persuade themselves. But there is always that little bit which they leave behind, and which they fondly hope will not grow because it is such a little bit. Alas! that so little leaven should leaven the whole lump! If one turns to the surgery books of a hundred and fifty or two hundred years ago, the true method of removing a cancerous breast will be found. The breast was laid hold of with great pincers, and having been cut clean off the surface was seared with a red hot cautery. Against a proceeding so shocking to the eye modern taste revolted, and so, for many years, surgeons have been removing a little elliptical bit of skin including the nipple, and have been carefully dissecting out the subjacent mamma. Then the remaining skin all impregnated with cancer germs has been carefully laid down again and neatly stitched together, so that everything should heal up quickly. Hence removal of a cancerous breast after this fashion came to be regarded as a comparatively slight operation. Very few people died as an immediate result of it,—very few indeed. Unfortunately, at a little later period, they all died from want of a little more of it; so that, looked at from another point of view, it was the most useless of all operations, inasmuch as it never effected a cure. My present contention, therefore, is for a return to the old plan of sweeping everything away and leaving a great hole, if you like."

He gives a synopsis of 46 cases, in 5 of which the breast alone was removed, while in 41 the breast was removed and the axillary glands cleared out.

6 cases proved fatal after operation;
11 had no reappearance of disease, and 10 died from it;
3 died from other causes under 2 years;
10 remained free from 2 to 10 years after operation;
5 " " 1 to 2 " "
9 done within last 12 months not reckoned;
1 lost sight of;
1 done for relief only.

### A Tape-worm Trap.

In the *New York Medical Record* (March 31, 1883) is an account of an invention patented by Alphaeus Myers, M.D., of Logansport, Ind., in 1854, for the purpose of trapping tape-worms. The instrument is of gold an inch long and one-fourth of an inch in diameter, and has an elaborate interior economy. After being baited with a tempting morsel and attached to a string the trap is swallowed. The unwary worm pushes in its head, is caught and dragged out by the string. To give completeness to the record the number of the patent, 11,942, is given. The inventor found the plan successful!

### Removal of Gall-bladder.

From a patient who, for six years, had suffered much from repeated attacks of biliary colic Dr. Langenbuch, in November, 1882, extirpated the gall-bladder. A T-shaped incision was made at the outer margin of the rectus muscle; the cystic duct was ligatured, the bile removed by aspiration, and the bladder cut away. The patient made a very rapid recovery, having got up in twelve days, and gained thirty pounds in weight in six weeks. At the end of four months he reported himself as being in perfect health.—*Berliner Klinische Wochenschrift.*

### Gas-light from Fæces.

The problem of the utilisation of sewage has received a novel solution at the hands of an ingenious German. By the decomposition of human fæces a light-yielding gas is obtained, which, *after purification*, may be used for ordinary domestic purposes. *Der Technicker*, which gives an account of the process, says that a Breslau hotel has been successfully lighted by this means.

### Rupture of the Aorta during Parturition.

The case was that of a woman of thirty-eight, apparently in good health, the os was dilating naturally, and the progress of labour seemed normal. Suddenly she was seized with convulsious and died collapsed. The forceps was applied and a living child delivered. At the autopsy a rupture of the aorta half an inch above the aortic valves was found. The pericardium was distended with blood.—*Centralblatt für Gynäkologie.*

### Surgical Dilation of the Pylorus.

In an individual suffering from pyloric stenosis from a cicatrix, Professor Loreta of Bologna, after having made an incision in the epigastrium, and opened the stomach, mechanically dilated the pylorus. The result was most successful, since, on the seventh day, the phenomena caused by the stenosis had disappeared, and the patient was going on well in every way.—*London Medical Record.*

### The Results of Resections of the Pylorus for Cancer, as given by Rydygier, are as follows:—

Sixteen surgeons have operated upon 23 cases, all but two of which have been cancer. Of the last two operations one was performed by Rydygier in a case of stenosis, caused by round ulcer, which terminated successfully; and the other by Lanenstein, in a case of supposed cancerous tumour, which at the autopsy proved to be one of gangrene of the transverse colon. Of the 23 cases, 19 proved fatal; viz., 15 some hours after operation, 3 on the seventh or eight day, and 1 (Billroth's) four months after from relapse. Of the 4 recoveries, 1 belongs to Billroth (no relapse having occurred in six months), 1 to Wolfer (the patient seeming well at the end of a year), 1 to Czerny (seven months without a relapse), and 1 to Rydygier.—*Medical Times and Gazette.*

### Cure of Squint without operation.

Dr. Boucheron maintains that cases of convergent strabismus are, in the early stages, before permanent contraction of the internal rectus has taken place, curable without operation. As convergence is caused by efforts of accommodation for near objects, paralysis of the accommodation by the constant use of mydriatics for two or three weeks will, in most cases, effect a cure. In eight out of nine cases of intermittent strabismus he obtained a cure in this way.—*Schmidt's Jahrbücher.*

### Scarlet Fever and Cerebro-Spinal Meningitis in Horses.

A writer in *The New York Times* comments on the claim of Dr. J. W. Stickler to have discovered a preventive of scarlet fever in the equine virus. He adds: "I have long known that scarlet fever exists among horses, and that it has been recognised, especially by French veterinary surgeons, but could obtain no information about it in New York, although I was well satisfied that it lurked among one of the forms of so-called "pink-eye," and I have several years ago called attention to this fact. It is well known also that the great epidemic of cerebro-spinal meningitis among horses in 1871 was followed by the greatest outbreak of that disease among our citizens in 1872. There is some well established connection between the two. I have long thought that scarlet fever and cerebro-spinal meningitis were carried by grooms and hostlers to their own homes, and perhaps to those of their masters and patrons, but could not positively prove the facts, because so much concealment and prevarication is always covered around such matters arising from ignorance and surprise at such notions, more perhaps than from deceit.—*New York Medical Record.*

### New Method of Treating Displacements of the Uterus.

For intractable cases of displacements of the uterus, chiefly retroversion, Dr. Alexander recommends an operation by drawing out the round ligaments through incisions made over the inguinal openings and fixing them there. He speaks of ten cases so operated on. Except in one case, where matters were made worse by the substitution of an anteversion for a retroflexion, good results were got in all. The operation is thus performed. The pubes being shaved, the pubic spine is felt with the finger, and an incision made upwards and outwards for two or three inches in length in the direction of the inguinal canal. The incisions are carried downwards till the vertical fibres that cross the external abdominal ring are exposed. These are cut through, and the terminations of the ligaments are seen bulging outwards as a reddish white tissue. This is raised by an aneurism needle, caught by the fingers and dragged out. On traction the thick round ligament appears, and by vaginal examination will be felt to draw the uterus along with it. This being done on both sides the ends of the ligaments are fixed by catgut ligatures at their point of exit from the abdominal wall. The uterus should be placed in position by the sound and not by traction on the ligaments. A pessary ought to be worn for some weeks after operation.—*Liverpool Med. Chir. Journal*, January, 1883.

This ingenious operation was conceived and executed contemporaneously and independently by Dr. James A. Adams, Demonstrator of Anatomy in the Glasgow University, and by Dr. Alexander.

[The Editor performed this operation in a bad case of retroversion and retroflexion after every other means had failed. It kept the uterus in position and relieved the symptoms for a very few months; but matters gradually got as bad as ever. As this proceeding in no way changes the texture of the portions of round ligaments left, it seems not unnatural that traction should succeed in causing their elongation after, just as it did before, operation. Further reports are necessary to show that the good results have been permanent in Dr. Alexander's cases.]

